# Factors Associated with Food Insecurity in Latin America and the Caribbean Countries: A Cross-Sectional Analysis of 13 Countries

**DOI:** 10.3390/nu14153190

**Published:** 2022-08-03

**Authors:** Akram Hernández-Vásquez, Fabriccio J. Visconti-Lopez, Rodrigo Vargas-Fernández

**Affiliations:** 1 Centro de Excelencia en Investigaciones Económicas y Sociales en Salud, Vicerrectorado de Investigación, Universidad San Ignacio de Loyola, Lima 15024, Peru; 2 Department of Health Sciences, Universidad Peruana de Ciencias Aplicadas, Lima 15023, Peru; u201712069@upc.edu.pe; 3 Facultad de Ciencias de la Salud, Universidad Científica del Sur, Lima 15067, Peru; jvargasf@cientifica.edu.pe

**Keywords:** food insecurity, COVID-19, Latin America, Caribbean region, cross-sectional studies

## Abstract

It is estimated that Latin America and the Caribbean (LAC) is the region with the second highest figures for food insecurity (FI) globally, with a prevalence of 40.9% in the entire region. This cross-sectional study analyzes the household factors associated with FI across 13 LAC countries. We used data from the first round of high-frequency phone surveys, conducted by the World Bank. Approximately 4 out of 10 people in LAC experienced FI during the first phase of the COVID-19 pandemic. FI was positively associated with the number of individuals aged from 5 to 18 years, the number of men, the illness, accident, or death of an income-earning household member, and health expenditure due to COVID-19 or other illnesses, as well as the increase in food prices, reduced family income, and job loss by a member of the household. On the other hand, households located in capital cities and those with more bedrooms were less likely to have experienced FI. The design of social policies must focus on the economic deficiencies experienced by the LAC population, with unemployment, reduced income, and high food costs being the main factors that must be addressed to ensure adequate nutrition.

## 1. Introduction

Food insecurity (FI) is a public health concern in various regions of the world and is defined as the lack of constant access to enough food for an active and healthy life and to avoid the risk of suffering from nutritional disorders and other related diseases [1]. Although FI is on the agenda of the Sustainable Development Goals (SDGs) to end hunger and all forms of malnutrition by 2030 [2], it is estimated that 1955.9 million people were living with FI globally in 2019, and this number was raised to 2297.8 million (an increase of 341.9 million compared to 2019) due to the COVID-19 pandemic in 2020 [3]. This increase can be attributed to the preventive measures taken by the government institutions of the countries, resulting in higher unemployment rates, salary cuts, increases in food prices due to lack of production and distribution, and the inability to execute strategies or social programs to mitigate hunger and malnutrition [4,5].

In the Latin American and Caribbean (LAC) region, the preventive measures taken by governments have exacerbated FI, increasing the prevalence of poverty, social inequalities, vulnerability to natural disasters, and low economic growth [6]. It is estimated that LAC is the region with the second highest figures for FI globally, with a prevalence of 40.9% in the entire region (compared to North America and Europe, with 8.8%) in 2020. It is also one of the regions in the world presenting the highest FI growth rate (from 22.9% in 2014 to 31.7% in 2019) [7,8]. These LAC figures can be influenced by various factors at the individual level and food context, such as gender, level of education, age, the number of members of the household, area of residence, economic income of the household, government support payments, job loss, or job change [9,10,11,12,13]. Another critical factor in FI is agriculture, since this sector plays a strategic role in improving food availability [14]. It is estimated that the promotion of investments in agricultural infrastructure and extension services, together with the adoption of measures aimed at increasing the purchasing power of households, especially those in rural areas, are key factors in improving the availability of, and access to, food [15]. With the COVID-19 pandemic, LAC governments implemented some measures to counteract poverty, such as social assistance programs, the direct payment of bonuses, unemployment benefits, and food donations [13].

The LAC region is characterized as being composed of low- and middle-income countries, in which the proportion of people’s budget spent on food is higher compared to high-income countries [5], and a minimal calorie diet is more expensive compared to the global average (USD 1.06 vs. USD 0.54 per day) [16]. Moreover, in the LAC region, there are income disparities, inequalities in the opportunities to access goods and public services, as well as high rates of poverty, unemployment, and high inflation [17]. All of this was enhanced by the arrival of COVID-19, with an estimated 9.1% decrease in the gross domestic product of LAC in 2020 [6]. In this scenario, inequality represents a major obstacle to the economic growth and the achievement of the SDGs in LAC countries, since it affects access to food and thereby contributes to FI.

A recent study using information from the high-frequency phone surveys (HFPS) of the World Bank database reported that the prevalence of FI in LAC countries was high at the beginning of the COVID-19 pandemic and had progressively declined by the end of the year 2020 [18]. However, the studies on the state of FI show little evidence of the availability of resources, the increase in food prices, and other factors that affect the existing reality in this region [9,19,20,21]. In this article, we aimed to analyze the household factors associated with FI across 13 LAC countries.

## 2. Materials and Methods

### 2.1. Data

This cross-sectional study used data from the HFPS conducted by the World Bank. The HFPS collected information on changes in employment and income, food insecurity, and access to health, education, and finance, among other subjects. The HFPS followed a panel format over three rounds of data collection in 12 countries and four rounds in Ecuador. The first round was conducted between 8 May and 14 June 2020, the second round from 5 June until 16 July, and the third was from 5 July until 25 August 2020. In Ecuador, the fourth round was collected between 15 and 25 August 2020 [22,23]. This study used the information exclusively from the first round.

The sample is based on a dual framework of cellphone and landline numbers generated through a random digit dialing (RDD) process. The RDD approach produces all possible phone numbers in the country under the national phone numbering plan and draws a random sample of numbers. This method ensures the coverage of all landline and cellphone numbers active at the time of the survey. The HFPS has two sampling units: households and individuals. Sampling weights were computed for each unit and were used accordingly to obtain estimates of interest for each unit. Further methodological details are reported elsewhere [22,23].

### 2.2. Setting and Participants

The HFPS was applied to households from 13 countries in LAC: Argentina, Bolivia, Chile, Colombia, Costa Rica, the Dominican Republic, Ecuador, El Salvador, Guatemala, Honduras, Mexico, Paraguay, and Peru. Respondents eligible for the HFPS were adults who were 18 years old and above. Only one respondent per household was interviewed, and the participant answered both individual and household-level questions [22,23].

### 2.3. Measures

FI (yes/no) was defined as the main outcome of interest and was measured with the following questions: Has your household run out of food due to lack of money or other resources? (yes/no); Were you or any other adult in your household hungry but not eating because there was not enough money or other resources to feed yourself? (yes/no); Did you, or any other adult in your household, have to “skip” a meal due to lack of money or other resources? (yes/no); Did you, or any other adult in your household, go without food for an entire day due to lack of money or other resources? (yes/no). The selection of the questions was based on the Food and Agriculture Organization Food Insecurity Experience Scale for households [24]. If the answer “yes” was given to any of the previous questions, then the individual was considered to have experienced FI.

Based on previous studies using the HFPS [25,26,27], we included the following predictors: the location of the household (non-capital/capital), number of bedrooms, number of individuals aged between 5 and 18 years, number of male individuals in the household, the illness, accident, or death of an income-earning household member (yes/no), health expenditure associated with COVID-19 or another illness (yes/no), rising prices of food consumed in the household (yes/no), family income during the pandemic (increased/remained equal/decreased), and whether a household member received cash, a check, or transfer from a government institution, non-government institutions, companies, or religious institutions (yes/no).

### 2.4. Statistical Analysis

All the statistical analyses were performed using Stata v16.0 (StataCorp, College Station, TX, USA). All the descriptive analyses were performed while taking the complex survey design into account to capture variations due to the weighting of the HFPS. The demographic characteristics of the population included were described using absolute and relative frequencies. The prevalence of FI, including the combinations of the four questions that were used measured FI, were described using absolute and relative frequencies.

Generalized linear mixed-effect (GLME) models with the Poisson family and logarithmic link were estimated to examine the association between FI and the predictors, grouped according to the countries. To run the models, the weighting of the HFPS was escalated following the recommendations of Adam Carle [28]. Afterward, we estimated the bivariate model to assess the associations between the outcome and each predictor individually. We then ran a multivariable GLME, which included predictors that yielded a *p*-value < 0.20 in the bivariate analysis. Lastly, we performed a second adjusted multivariate GLME, which included an interaction term of the job loss and the receiving of cash, a check, or a transfer. The results of the bivariate and multivariate analyses were presented in terms of prevalence rates (PR) and adjusted prevalence rates (aPR) along with their 95% confidence intervals (CI). We also checked the variation inflation factors (VIF) to detect multicollinearity among the predictors in the multiple regression model. The VIF was <1.41 for all the variables entered in the model, which indicates the absence of multicollinearity among the covariates [29].

## 3. Results

**Figure 1:**
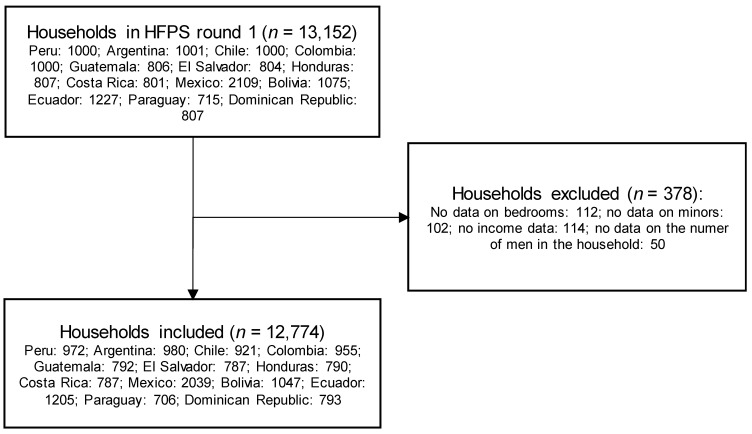
Flowchart of the selection of adults included in the study. HFPS: high-frequency phone surveys.

The study flowchart is shown in Figure 1. In round one, 13,152 households were surveyed. The sample size ranged from 715 in Paraguay to 2109 in Mexico. After dropping 378 observations due to missing information, we finally included 12,774 observations in the analysis.

Table 1 reports the characteristics of the households included in the analysis according to the different countries. Households in Mexico, Bolivia, Ecuador, and Chile were mostly located in the capitals, while households located in Guatemala, El Salvador, Costa Rica, and Paraguay were mostly located in non-capital cities. Households in Argentina had the lowest mean number of individuals aged between 5 and 18 years (0.7), and Guatemala reported the highest mean (1.7). In Ecuador and the Dominican Republic, 6.2% and 5% of the households, respectively, reported that an income-earning household member had suffered an illness, accident, or death. In Ecuador, Peru, and the Dominican Republic, the households reported the highest frequency of health expenditure due to COVID-19 or another illness. Except for the households in Costa Rica, in all the countries, more than 70% of households reported an increase in food prices. More than 8 out of 10 households in Peru reported a reduction in the family income, while for Argentina the frequency was 4 out of 10 households. Job loss by a household member was reported by 73% of Peruvian households, and 22% of Argentinian households. In Bolivia, 37% of the households reported receiving cash, a check, or a transfer from government institutions, non-government institutions, companies, or religious institutions.

According to the definition used in this study, FI was reported in 39% of the households (Table 2). However, we assessed different definitions of FI to explore the prevalence of FI. For instance, when we applied the strictest criteria (meaning that a household responded yes to all four of the questions), the prevalence dropped to 7.8%. For the remaining combinations, the prevalence ranged from 8% up to 32%.

The factors associated with FI are reported in Table 3. Individually, in the bivariate model, all the predictors were associated with FI, except for the receiving of cash, a check or a transfer, which yielded an association at a 0.1 level of significance. In the adjusted model, we found that households located in the capital cities had a lower probability of experiencing FI (aPR: 0.82, 95% CI: 0.77 to 0.86). FI was inversely associated with the number of bedrooms (aPR: 0.84, 95% CI: 0.81 to 0.87), but positively associated with the number of individuals aged between 5 and 18 years (aPR: 1.07, 95% CI: 1.05 to 1.10) and with the number of males in the household (aPR: 1.04, 95% CI: 1.03 to 1.05). Moreover, FI was positively associated with the illness, accident, or death of any income-earner in the household (aPR: 1.13, 95% CI: 1.02 to 1.25), health expenditure due to COVID-19 or another illness (aPR: 1.21, 95% CI: 1.12 to 1.30), rising prices of food consumed in the household (aPR: 1.54, 95% CI: 1.38 to 1.71), reduced family income (aPR: 1.27, 95% CI: 1.03 to 1.55), and with job loss by any member of the household (aPR: 1.71, 95% CI: 1.57 to 1.86). In the multivariate analysis, we found no association between FI and the receiving of cash, a check, or a transfer (*p* = 0.640). Lastly, when assessing the interaction term of job loss and the receiving of cash, a check, or a transfer, we found that FI was lower in households which reported the job loss of any household member and received any cash, a check, or a transfer compared to other households that did not meet these criteria (aPR: 0.84, 95% CI: 0.71 to 0.99).

## 4. Discussion

This study was based on the World Bank HFPS and aimed to determine the prevalence and factors associated with FI in 13 LAC countries. In general, the LAC countries included in the analysis have common characteristics or patterns that have made it possible to address FI during the COVID-19 pandemic [30,31]. For instance, LAC countries have experienced a nutrition transition in recent years that is characterized by changes in dietary patterns, eating habits, and lifestyles due to increased urbanization and economic growth [30]. During the pandemic, in LAC, public health measures such as confinement have generated a decrease in agricultural activity and an increase in food prices in most countries, which caused a change in eating behaviors and inequalities in nutrition [31]. FI was reported in 39% of households surveyed in LAC based on only the first round of the survey. FI was positively associated with the number of individuals aged from 5 to 18 years, the number of men, the illness, accident, or death of an income-earning household member, and health expenditure due to COVID-19 or other illness, as well as the increase in the prices of food consumed, reduced family income, and job loss by any member of the household. On the other hand, households located in capital cities and those with more bedrooms were less likely to have experienced FI.

Approximately 4 in 10 households surveyed in the 13 LAC countries included were reported as having experienced FI during the COVID-19 pandemic. This finding is higher than that reported for high-income countries, such as the United States (where the FI rate ranged between 10.8% and 73.9%), [32], Australia (11.8%) [33] and Portugal (6.8%) [34], while in low- and middle-income countries, such as Bangladesh (90.6%) [35], Ethiopia (38.4% in rural households and 48.3% of urban households) [27], India (62%) [36], and Jordan (40.7%) [37], a similar or even lower pattern was observed because the COVID-19 pandemic affected low- and middle-income countries more in health and economic terms [38]. Another study, which was based on the implementation of a survey through a social network and conducted in 20 Latin American countries during the first wave of the COVID-19 pandemic, reported a higher prevalence (75.7%) compared to our findings [9]. This difference could be based on the use of a virtual survey (which has limitations in methodology and execution) [39], different levels of analysis (individual level versus household level), and a different operational definition of FI compared to our study.

The high prevalence of FI identified in our study can be attributed to the fact that the COVID-19 pandemic generated greater food shortages due to social confinement measures, leading to a reduction in food cargo and transportation services during the pandemic [40,41,42]. This problem induced low production (a shortage of labor due to the fear of virus transmission), and difficulties in transportation, with subsequent delays in the delivery chains of goods and services, which can cause shortages and the low marketing of foods in the market. Likewise, there was an increase in the price and a decrease in the quality of food due to import restrictions, the closure of restaurants and low-budget retail markets, and the cessation of family-based agricultural production and marketing, and an increase in poverty due to the high rates of unemployment and informal work [40,41,42]. These consequences of the pandemic have made unhealthy foods (foods with a high content of saturated fats and added sugars) the cheapest alternatives for families in LAC due to the increase in the prices of foods with a high nutritional content (fruits, vegetables, and legumes), which has had a negative impact on the nutritional quality of the diet and contributed to the double burden of malnutrition [43,44,45]. In this sense, government institutions that prioritize strategies for reducing hunger and its consequences on health must consider the social barriers generated by the pandemic to guarantee that food transportation and delivery chains are not interrupted.

Likewise, it was found that FI was positively associated with the number of individuals aged from 5 to 18 years, the illness, accident, or death of an income-earning household member, and health expenditure due to COVID-19 or another illness, as well as the increase in the prices of food consumed, reduced family income, and job loss by any member of the household. These factors that were associated with a higher prevalence of FI have been observed in studies conducted in other geographical contexts, such as Ethiopia [27], Uganda [25,46], Kenya [46], Bangladesh [35], Australia [12], and other countries belonging to the sub-Saharan African region [26]. These findings can be attributed to the main effects of the COVID-19 pandemic on various socioeconomic aspects of the populations of low- and middle-income countries. Specifically, one of the main effects of the pandemic on food security was its impact on employment, with job opportunities having decreased due to the difficulties of enabling personal interactions between workers in sectors such as construction, manufacturing, and small local businesses [47,48]. In addition, most low- and middle-income countries have a high percentage of informal work that can generate a higher unemployment rate due to the prevalence of short-term contracts in that sector. These consequences on employment are expressed by the low economic income and the financial status of the household [47,48].

Another effect of the pandemic was the increase in the cost of food, which caused a greater demand for foods with little nutritional content, such as sugary drinks and salty and high-fat foods, due to disruptions in the supply chains that deprived fresh and healthy food stocks [46,49,50]. On the other hand, our study found that the number of men in the household was positively associated with the presence of FI [49,51,52,53]. During 2020, a difference in labor participation according to sex was reported in LAC (69% for men vs. 46% for women) [54]. This difference could explain our findings, since households with a greater number of men may have been more economically affected by greater job losses.

We reported that households located in capital cities and those with a higher number of bedrooms were less likely to have experienced FI. In general, various studies have demonstrated that households located in rural areas had a higher prevalence of FI compared to those located in urban areas, since rural households were most affected by the impact of the pandemic in economic, financial, and social terms [55,56]. A pattern such as that found in our study was observed in LAC countries, with rural households presenting with a higher prevalence of FI. This pattern can be attributed to the fact that the populations in these areas have higher rates of informal employment, live in conditions of extreme poverty, and have greater difficulties in distributing and transporting their agricultural products due to a lack of workers and an increase in transport prices [19]. On the other hand, households located in urban areas such as cities have greater access to social programs to mitigate the financial crisis and food shortages [10]. In addition, it was found that households with a higher number of bedrooms in the home were less likely to have experienced FI, which is likely attributable to the socioeconomic level of the household. Specifically, poor households experienced a negative impact on food security during the pandemic due to their low incomes, reduced incomes, or the fact that they were running out of savings, making it difficult to access foods with the highest nutritional content due to their high cost [19,20,36,57]. Government institutions in LAC must reinforce hunger mitigation strategies, prioritizing poor households located in rural areas to improve their nutritional status and prevent diseases related to nutritional deficiencies, even more so when we consider that these households already had a high proportion of FI before the pandemic.

Our findings could have an impact on the future public policies carried out in LAC countries. During the pandemic, LAC countries established preventive measures to restrict the mobility of people, leading to a significant economic reduction in this region. To address this economic crisis, most LAC countries executed new social programs and improvements in the supply chain. One of the most relevant measures were cash transfers or emergency bonds to alleviate the economic problems of the poorest households. The coverage and size of social benefits was not similar across all countries. In countries such as Mexico, Trinidad and Tobago, and Costa Rica, payments or coverage were not extended [58]. In this sense, our findings could help government institutions in LAC countries to improve the prioritization of the measures that were adopted during the pandemic, of which the greatest beneficiaries should be households with a high unemployment rate, those with children, and those which are located in the rural and poorer areas of this region. In this way, not only would the economic problem be addressed, but so would the repercussions on the health of individuals through the mitigation of nutritional deficiencies due to a lack of access to foods with a high nutritional content.

This study has several limitations to note. The cross-sectional design of the study does not allow for the assessment of causality between the independent variables and FI due to the lack of temporality in its measurements. Additionally, the data reported by the respondents may be subject to memory bias or social desirability because they concern specific events that occurred at the time of a health and economic crisis. Moreover, telephone data collection limits the depth and potential of data collection due to the complexity of the questions, which tends to reduce the quality of the data and result in an uneven coverage, especially in areas with low network coverage or electricity. Despite these limitations, the HFPSs were conducted by the World Bank during the COVID-19 pandemic to collect socioeconomic data from 100 countries and to assess changes in people’s well-being and vulnerability due to the impacts of the pandemic. Likewise, the HFPS sought to collect information in the first phase of the pandemic through telephone surveys. Due to the restrictions in mobility, telephone surveys were one of the most suitable ways of conducting interviews compared to other means, such as text messages or email [59].

## 5. Conclusions

In conclusion, approximately 4 out of 10 people in LAC experienced FI during the first phase of the COVID-19 pandemic. The number of individuals aged from 5 to 18 years old, the number of men, the illness, accident, or death of an income-earner, health expenditure due to COVID-19 or another illness, the increase in the prices of foods consumed, reduced family income, and job loss by any household members were positively associated with FI. The design of social policies must focus on the economic deficiencies experienced by the LAC population, with unemployment, reduced income, and high food costs being the main factors that must be addressed to ensure adequate nutrition.

## Figures and Tables

**Table 1:** Characteristics of the households included in the analysis by country.

Characteristics of the Household	All Countries	Peru	Argentina	Chile	Colombia	Guatemala	El Salvador	Honduras	Costa Rica	Mexico	Bolivia	Ecuador	Paraguay	Dominican Republic
	%	%	%	%	%	%	%	%	%	%	%	%	%	%
Overall (*n*)	12.774	972	980	921	955	792	787	790	787	2039	1047	1205	706	793
Location of the household														
Non-capital	47.2	56.6	63.3	48.7	64.1	70.9	76.9	64	73.4	24.8	27.8	39.4	70.9	66.7
Capital	52.8	43.4	36.7	51.3	35.9	29.1	23.1	36	26.6	75.2	72.2	60.6	29.1	33.3
Mean of bedrooms (SD)	2.3 (1.0)	2.3 (1.3)	2.2 (0.6)	2.6 (1.1)	2.4 (0.7)	2.4 (1.7)	2.1 (1.9)	2.3 (2.1)	2.5 (1.9)	2.3 (0.7)	2.4 (2.1)	2.2 (1.4)	2.6 (2.3)	2.3 (1.2)
Mean number of individuals aged between 5 and 18 years (SD)	1.2 (1.3)	1.3 (1.3)	0.7 (0.9)	0.9 (1.2)	1.1 (0.8)	1.7 (2.1)	1.2 (2.1)	1.6 (2.8)	1.0 (2.4)	1.2 (0.9)	1.5 (2.3)	1.1 (1.5)	1.3 (2.6)	1.2 (1.8)
Mean number of male individuals (SD)	1.9 (1.2)	2.2 (1.3)	1.4 (0.8)	1.7 (1.2)	1.9 (0.8)	2.3 (1.8)	1.9 (1.9)	2.1 (2.5)	1.8 (2.4)	1.9 (0.8)	2.4 (2.4)	2.1 (1.7)	2.0 (2.4)	1.9 (1.6)
Illness, accident, or death of an income-earning household member *														
No	96.8	95.5	98.5	95.5	97.0	96.0	95.2	95.8	97.9	97.1	97.9	93.8	97.3	95.0
Yes	3.2	4.5	1.5	4.5	3.0	4.0	4.8	4.2	2.1	2.9	2.1	6.2	2.7	5.0
Health expenditure associated with COVID-19 or another illness *														
No	90.9	88.4	94.3	90.3	91.4	92.5	91.7	93.4	93.8	90.5	90.6	84.9	94.5	90.7
Yes	9.1	11.6	5.7	9.7	8.6	7.5	8.3	6.6	6.2	9.5	9.4	15.1	5.5	9.3
Rising prices of food consumed in household *														
No	22.0	11.7	19.5	26.9	17.7	25.1	26.2	21.4	54.6	23.5	27.6	19.1	24.2	29.8
Yes	78.0	88.3	80.5	73.1	82.3	74.9	73.8	78.6	45.4	76.5	72.4	80.9	75.8	70.2
Family income *														
Increased	3.4	1.0	9.2	5.1	3.3	3.3	2.8	2.7	2.5	1.5	4.0	1.2	2.9	7.3
Remained equal	33.5	16.8	49.6	39.1	24.6	25.3	28.0	28.2	33.8	37.7	24.6	24.7	31.6	32.6
Reduced	63.1	82.1	41.2	55.8	72.1	71.4	69.3	69.2	63.7	60.8	71.4	74.2	65.4	60.1
Job loss by a household member *														
No	57.4	27.1	78.0	59.4	39.2	48.2	46.1	49.6	57.3	64.1	59.8	63.0	59.3	65.2
Yes	42.6	72.9	22.0	40.6	60.8	51.8	53.9	50.4	42.7	35.9	40.2	37.0	40.7	34.8
Household member received cash, a check, or transfer **														
No	90.5	84.9	90.1	91.6	88.3	96.7	72.3	98.0	85.4	96.0	62.8	95.5	70.8	90.5
Yes	9.5	15.1	9.9	8.4	11.7	3.3	27.1	2.0	14.6	4.0	37.2	4.5	29.2	9.5

* Since the beginning of the quarantine. ** From the beginning of the quarantine, by governmental institutions, non-governmental institutions, religious organizations, or companies. Percentages (%) are indicated unless otherwise specified. The sample weights of each survey are included.

**Table 2:** Characteristics of the households included in the analysis by questions of food insecurity.

Question(s)	All Countries% (95% CI)
Q1	32.7 (31.4–34.0)
Q2	24.3 (23.1–25.5)
Q3	27.8 (26.6–29.0)
Q4	10.3 (9.5–11.2)
Q1 and Q2	21.1 (19.9–22.2)
Q1 and Q3	22.7 (21.6–23.9)
Q1 and Q4	9.3 (8.5–10.2)
Q2 and Q3	20.1 (19.0–21.2)
Q2 and Q4	8.8 (8.1–9.7)
Q3 and Q4	9.1 (8.4–10.0)
Q1 and Q2 and Q3	18.0 (17.0–19.1)
Q2 and Q3 and Q4	8.1 (7.4–8.9)
Q1 and Q2 and Q4	8.4 (7.6–9.2)
Q1 and Q3 and Q4	8.5 (7.7–9.3)
Q1 and Q2 and Q3 and Q4	7.8 (7.1–8.6)
Q1 or Q2 or Q3 or Q4	39.2 (37.9–40.6)

Q1: Has your household run out of food due to lack of money or other resources? Q2: Were you or any other adult in your household hungry but not eating because there was not enough money or other resources to feed yourself? Q3: Did you, or any other adult in your household, have to “skip” a meal due to lack of money or other resources? Q4: Did you, or any other adult in your household, go without food for an entire day due to lack of money or other resources? The sample weights of each survey are included.

**Table 3:** Factors associated with food insecurity (*n* = 12,774).

	Bivariate Model		Adjusted Model 1		Adjusted Model 2	
Variable	PR (95% CI)	*p*-Value	aPR (95% CI)	*p*-Value	aPR (95% CI)	*p*-Value
Location of the household						
Non-capital	Ref.		Ref.		Ref.	
Capital	0.75 (0.69–0.80)	<0.001	0.82 (0.77–0.86)	<0.001	0.82 (0.77–0.86)	<0.001
Number of rooms	0.84 (0.80–0.89)	<0.001	0.84 (0.81–0.87)	<0.001	0.84 (0.81–0.87)	<0.001
Number of individuals aged 5 to 18 years	1.12 (1.09–1.15)	<0.001	1.07 (1.05–1.10)	<0.001	1.07 (1.06–1.10)	<0.001
Number of individual men	1.07 (1.05–1.09)	<0.001	1.04 (1.03–1.05)	<0.001	1.04 (1.03–1.05)	<0.001
Illness, accident, or death of an income-earning household member*						
No	Ref.		Ref.		Ref.	
Yes	1.38 (1.22–1.57)	<0.001	1.13 (1.02–1.25)	0.022	1.13 (1.03–1.25)	0.025
Health expenditure associated with COVID-19 or another illness *						
No	Ref.		Ref.		Ref.	
Yes	1.38 (1.27–1.49)	<0.001	1.21 (1.12–1.30)	<0.001	1.21 (1.12–1.30)	<0.001
Rising prices of food consumed in household *						
No	Ref.		Ref.		Ref.	
Yes	1.98 (1.75–2.24)	<0.001	1.54 (1.38–1.71)	<0.001	1.54 (1.38–1.71)	<0.001
Family income e *						
Increased	Ref.		Ref.		Ref.	
Remained equal	0.79 (0.63–0.99)	0.041	0.89 (0.73–1.09)	0.273	0.90 (0.74–1.10)	0.308
Reduced	1.53 (1.23–1.89)	<0.001	1.27 (1.03–1.55)	0.023	1.27 (1.03–1.56)	0.022
Job loss by a household member *						
No	Ref.		Ref.		Ref.	
Yes	2.16 (1.92–2.43)	<0.001	1.71 (1.57–1.86)	<0.001	1.75 (1.59–1.93)	<0.001
Household member received cash, a check, or transfer ¶						
No	Ref.		Ref.		Ref.	
Yes	1.14 (0.99–1.31)	0.073	1.03 (0.91–1.16)	0.640	1.16 (0.96–1.40)	0.127
Job loss # Transfers						
Yes # Yes					0.84 (0.71–0.99)	0.043

* Since the beginning of the quarantine. ¶ From the beginning of the quarantine, by governmental institutions, non-governmental institutions, religious organizations, or companies. # Interaction. Estimates include the weights using method A for scaling the weights recommended by Carle. PR values were estimated from multivariable generalized linear models that additionally included random effects for the country.

## Data Availability

Not applicable.
